# International collaboration to develop a dental implant standard set: study protocol for a multicentre prospective cohort study for the validation of a core outcome set

**DOI:** 10.1136/bmjopen-2025-108369

**Published:** 2026-09-11

**Authors:** Milton Chin, Justin Pijpe, Juan Blanco, Marco Cune, Eppo Wolvius, Lea Kragt

**Affiliations:** 1 Department of Maxillofacial Surgery, Special Dental Care and Orthodontics, Erasmus University Medical Center Rotterdam, Rotterdam, Zuid-Holland, The Netherlands; 2 Department of Surgery and Medical-Surgical Specialties (Stomatology), Universidade de Santiago de Compostela, Santiago de Compostela, Spain; 3 Department of Oral Maxillofacial Surgery, Prosthodontics and Special Dental Care, St Antonius Hospital, Nieuwegein, Utrecht, The Netherlands; 4 Department of Restorative Dentistry, University Medical Centre Groningen, Groningen, Groningen, The Netherlands

**Keywords:** Dental Implants, Patient Reported Outcome Measures, Surveys and Questionnaires

## Abstract

**Introduction:**

Dental implants are a widely accepted solution for tooth replacement, but standardised outcome assessment, particularly patient-reported outcomes, remains limited. Moreover, international variability in implantology practices underscores the need for consistent and comprehensive evaluation tools. This study aims to develop and validate the DEntal implaNt Set (DENS), a standardised tool to capture clinician and patient perspectives on peri-implant health and restorative treatment outcomes.

**Methods and analysis:**

This prospective multicentre cohort study aims to include 1000 patients aged 18–75 years from 30 dental practices in the Netherlands and Spain. The DENS was developed by an international multidisciplinary team through structured iterative working sessions, grounded in the mandatory outcome domains of the Implant Dentistry Core Outcome Set and Measurement (ID-COSM) framework and informed by expert consultation with the ID-COSM authors. Patient-reported outcome measure (PROM) items were adapted from validated instruments and subjected to forward-backward translation into Dutch, English and Spanish. Data are collected at six time points from pre-implant placement to 12-month follow-up. Clinical outcomes and PROMs are assessed via the digital. Descriptive analyses and psychometric testing of the DENS instrument are planned at the end of the data collection phase.

**Ethics and dissemination:**

Ethical approval was granted by committees in the Netherlands (Erasmus University Medical Center: MEC-2023-0688; Anna Hospital Eindhoven: 2024.076; Anna Hospital Geldrop: MEC-24.008; St. Antonius Hospital: R&D/Z24.070/DENS; University Medical Center Groningen: METc 2024/389) and Spain (CEI-SL 2024/122). Participants provide informed consent, and no procedures exceed standard clinical care. All data are pseudo-anonymised. Findings will be shared through publications, conferences and professional networks. The DENS tool will be made available for clinical practice and research purposes to promote standardised, evidence-based practice in implant dentistry.

**Trial registration number:**

NCT07337057.

**Strengths and limitations of this study**

- International standardisation uses the Implant Dentistry Core Outcome Set and Measurement framework that identifies essential outcome domains for standardised measurement instruments for both patient and clinician perspectives.
- Multilingual validation follows professional translation methodology.
- Longitudinal tracking of at least 12-month systematic follow-up.
- Limited scope covers only two countries with 12-month follow-up.
- Implementation challenges include research-engaged practice bias and complex questionnaire administration.

## Background and rationale

 Tooth loss represents a significant global health challenge affecting individuals across all age groups, from young adults to the elderly. The extent of tooth loss varies considerably, ranging from single-tooth defects to complete edentulism. Multiple aetiological factors contribute to tooth loss, including dental trauma, caries, periodontal disease, oral malignancies and other pathological conditions.^1^

Since its initial description in the 1960s, dental implant therapy has evolved to become the gold standard treatment for tooth replacement and in case of functional denture problems. Dental implants are currently used for single-tooth replacement with crowns, multiple-tooth replacement with bridges and in edentulous patients with full dentures supported by implants. The decision to place a dental implant following tooth loss depends on multiple factors, including cost, longevity, oral and general health and the availability of alternative treatment options. This decision-making process is guided by country-specific clinical guidelines and protocols, if available, which can result in variations in indications and treatment approaches in implantology across different settings and even across different practitioners.^1–3^

Most studies in implant dentistry focus on clinical outcomes, such as implant success and survival rates, presence of dental plaque, bleeding on probing (BOP), probing pocket depth, bone loss, peri-implantitis and aesthetic assessments such as the Pink Esthetic Score and the White Esthetic Score.^4–6^ However, limited attention has been paid to the patient’s perspective. Recently, there has been growing emphasis on patient-reported outcome measures (PROMs) in implantology. In response, the International Team for Implantology (ITI) has formed a working group dedicated to developing core domains from the patient perspective.^7^ Nevertheless, to date, no standardised methods or validated questionnaires exist to systematically assess implant outcome, either from the patient perspective or from the clinical standpoint.

A standardised set of PROMs and clinician-reported outcome measures (ClinROs) would enable the implementation of uniform research within the field of implantology. In developing such a set, it is essential to identify which domains and instruments are most relevant and clinically meaningful to implantology. Recently, Tonetti *et al* published a consensus statement that describes the core domains for developing a core outcome set in implant dentistry. This set focuses on both clinical outcomes and patient-reported outcomes.^8^ Although this set was originally developed for research purposes, the domains might be highly relevant in clinical practice too.

In developing and implementing such a set, it is essential to account for the previously discussed international differences in implantology practices and language. Therefore, establishing this set through international collaboration is crucial to ensure appropriate cross-cultural validation. Furthermore, it is important to investigate the extent to which these core domains can be applied in clinical practice using validated questionnaires targeting both patients and healthcare professionals. If this set proves to be implementable in clinical practice, it will constitute a significant advancement by facilitating the systematic evaluation of implant care, both in routine clinical application and in standardised research at national and international levels. A validated and reliable outcome set would support clinicians in assessing, monitoring, improving, differentiating and benchmarking their clinical performance.

## Aim

The aim of this protocol is to describe the development and implementation of the DEntal implaNt Standard set (DENS), a standardised outcome measurement instrument comprising ClinROs and PROMs, based on core-domains of the Implant Dentistry Core Outcome Set and Measurement (ID-COSM) framework, for evaluating peri-implant health and restorative outcomes in routine implant dentistry practice across two countries.

Formal psychometric validation of the DENS instrument is planned as a subsequent step following completion of data collection.

Following psychometric validation, the DENS instrument will be made available to support in clinical practice and research settings internationally.

## Material and methods

### Overall study design

The project is a prospectively designed multicentre cohort study and aims to include patients from 20 dental practices in the Netherlands (coordinated by Erasmus University Medical Center, Rotterdam) and Spain (coordinated by University of Santiago de Compostela). From the dental practices, 1000 consecutive patients are included within 24 months. ClinROs and PROMs are systematically collected at multiple timepoints throughout the treatment protocol, including baseline, implant placement, integration phase and restorative outcomes.

Patient enrolment commenced on 1 June 2024 in the Netherlands and on 1 October 2024 in Spain. Data collection is projected to be completed by 1 June 2028.

### Establishment of the research team

A multidisciplinary working group comprising implantologists, prosthodontists and researchers from the Netherlands and Spain was established to develop a comprehensive outcome measurement set. The international team represented three academic centres: Erasmus University Medical Center (Rotterdam, the Netherlands), the University of Santiago de Compostela (Madrid, Spain) and the University Medical Center of Groningen (Groningen, the Netherlands), ensuring diverse clinical perspectives and cultural considerations.

### Development of the preliminary DENS

The DENS was developed by an international multidisciplinary team, based on the five mandatory outcome domains established by Tonetti *et al* through the ID-COSM initiative.^8^ These domains were identified through a rigorous international consensus process comprising systematic reviews, patient focus groups and a three-round Delphi survey, providing the evidence-based foundation for standardised outcome measurement in implant dentistry.

Given that the ID-COSM mandatory outcome domains had already been established through an international consensus process, a new Delphi procedure for domain selection was not considered necessary. Instead, item selection and refinement were conducted through structured expert working sessions within the international research team.

Building on these domains, candidate questionnaire items were drawn from validated instruments: the Oral Health Impact Profile (OHIP-14), OHIP-EDENT, the Psychosocial Impact of Dental Aesthetics Questionnaire (PIDAQ), the Dental Satisfaction Questionnaire (DSQ) and the Patient Satisfaction Questionnaire (PSQ).^9–13^ Items were selected and refined through multiple iterative working sessions with the core research group across three academic centres and evaluated against three criteria: (1) clinical relevance to routine implant dentistry practice, (2) direct alignment with an ID-COSM mandatory outcome domain and (3) comprehensibility and respondent burden for both clinicians and patients. Items considered overlapping, redundant or insufficiently specific to the implant dentistry context were excluded through team consensus. The preliminary item set was subsequently presented to the authors of the ID-COSM report for expert review, and their feedback was incorporated into the final instrument.

Questionnaires were then professionally translated into Dutch, English and Spanish using forward-backward translation methodology to ensure linguistic and cultural equivalence across both study countries. The present multicentre prospective cohort study constitutes the implementation phase, during which the DENS is administered under clinical conditions. Formal psychometric validation, including assessment of internal consistency (Cronbach’s α), structural validity and construct validity in accordance with COnsensus-based Standards for the selection of health Measurement INstruments (COSMIN) methodology, is planned at the end of the data collection phase and will be reported in a separate publication.

### DENS domains

The ID-COSM report by Tonetti *et al*^8^ identified the core outcome domains essential for developing standardised measurement instruments, providing the foundational framework for both clinician-reported and patient-reported outcomes in implant dentistry. The current project adheres to these domains to enhance the consistency and quality of research, ultimately advancing evidence-based implantology and optimising patient care.^8^ The DENS will include both patient-reported outcomes and clinician-reported outcomes, based on the following key domains:

- Surgical morbidity and complications (until delivery of final prosthesis).
- Peri-implant tissue health status.
- Intervention-related adverse events (including implant/prosthesis loss—after delivery of final prosthesis).
- Complication-free survival (implant/prosthesis related).
- Overall patient satisfaction and comfort.

### Clinical-reported outcomes measures

The ClinROs include baseline patient characteristics. The following case-mix variables are collected: age, gender, oral hygiene practices (eg, tooth brushing frequency, interdental cleaning), dental visits in the past year, tobacco use status (current smoker, former smoker, non-smoker), alcohol consumption (daily vs occasional) and chronic medical conditions (eg, diabetes, hypertension).

In line with the mandatory domains of the ID-COSM, additional clinical variables are assessed at each visit. These include:

- Periodontal health, using the Periodic Periodontal Screening score at the implant site and neighbouring teeth.
- BOP.
- Occurrence and timing of complications following implant placement throughout the 12-month follow-up period.

At the time of implant placement, the following data are also collected:

- Implant details (brand, type).
- Type of implant-supported restoration, including:
  - Single-unit fixed dental prosthesis (screw-retained or cemented).
  - Multi-unit fixed dental prosthesis.
  - Removable partial denture.
  - Removable full denture.

### Patient-reported outcome measures

A structured questionnaire was developed to address these mandatory domains, with responses collected using a 5-point Likert scale. Following each dental visit, participants receive the questionnaire by mail. It includes questions related to oral health, quality of life, patient satisfaction and expectations regarding treatment outcomes.

The PROMS items are adapted from validated instruments, including:

- OHIP-14.^10^
- OHIP-EDENT.^9^
- PIDAQ.^12^
- DSQ.^11^
- PSQ.^13^

To ensure responses reflect each patient’s personal experience, questionnaires are completed individually via digital forms. The specific questions used in the patient-reported outcomes assessment are presented in box 1.

Box 1 **Patient-reported outcomes questions**

Patient questionnaire

1. Do you experience any impairment in your ordinary/daily life (total) activity (eg, work) since your last visit?
2. Do you experience pain related to your last visit?
3. Do you experience chewing difficulties because of your dentition?
4. Do you feel that your crown/bridge/prosthesis is fitting properly?
5. Do you have difficulties pronouncing any words (due to the procedure)?
6. Are you satisfied with the appearance of your crown/bridge/prosthesis?
7. Do you experience difficulties cleaning your crown/bridge/prosthesis?
8. Do you feel that life in general is less satisfying because of your dentition related to the surgical procedure?
9. How satisfied are you with the comfort throughout the treatment?
10. How satisfied are you with the overall treatment?
11. How satisfied are you with the practitioner/professional?

### Eligibility criteria for participating dental implant clinics and included patients

**Figure 1:**
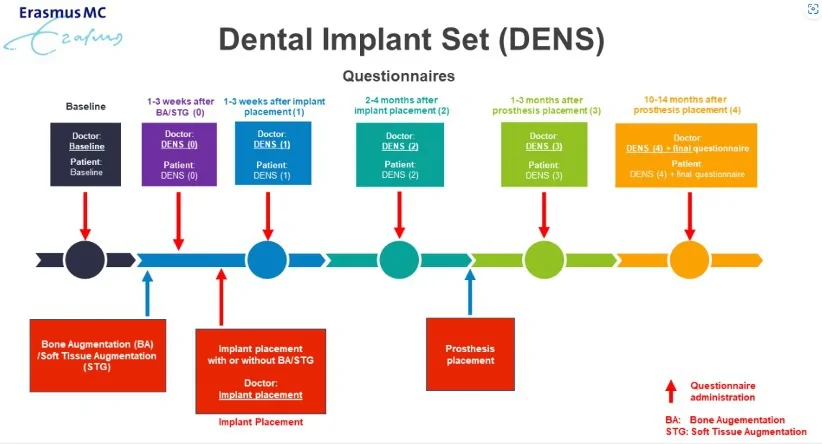
Overview of DENS follow-up.

For participating dental practices, it is essential to ensure that the centres adhere to the follow-up schedule (figure 1). Only practices able to ensure patient contact for at least 1–2 years post-implantation are included to guarantee sufficient follow-up and data completeness. Additionally, practices must remain informed about any complications that may arise during the placement of the suprastructure. Each practice is expected to include a minimum of 30 and a maximum of 50 patients in the study.

All patients aged 18–75 years who have an indication for implant-supported fixed single-tooth or multiple-tooth replacement or implant-supported removable prosthesis are eligible to be enrolled in this study by their treating clinician/dental practice. This study will also include patients that require minor bone augmentation or soft tissue grafts.

Inadequate oral hygiene, low digital literacy, major bone-augmentation procedures (extra-oral donor sites), pregnancy, a history of radiotherapy in the head and neck region and language barriers are reasons to exclude patients from the study population.

### Patient and public involvement

The development of DENS is grounded in a framework that was established with explicit patient involvement. The ID-COSM outcome domains, which form the basis of the DENS instrument, were identified through a rigorous international consensus process incorporating the perspectives of 31 people with experience of dental implants, who participated in focus groups across four countries. Patient representatives further contributed to both the three-round Delphi survey and the final consensus meeting.^8^

Within the current study, patients participate as research contributors by completing digital questionnaires at six time points throughout their treatment trajectory, capturing their experiences with implant-supported tooth replacement from pre-treatment through to 12-month follow-up. Participants are introduced to the study by their treating clinicians, who obtain written informed consent prior to inclusion. Study information and findings will be disseminated to participants through the study website (www.densresearch.com) on completion.

### Sample size

As this project is exploratory in nature, a formal statistical hypothesis and sample size calculation is not appropriate. It is anticipated that each participating dental implant practice will recruit between 30 and 50 patients during the data collection phase of the project, with an expected total of 15 participating practices per country. This is expected to result in a total inclusion of approximately n=900–1500 patients. However, assuming a response rate of 70%, the final study population at the end of the follow-up period is estimated to be between n=630 and 1050.^14^ This sample size across multiple countries and diverse practice settings is expected to provide adequate representation of the target population for robust validation of the outcome measurement instruments.

### Data collection procedure

The questionnaires are administered to the clinicians and patients linked to the specific appointment dates.

Data are collected at multiple time points as suggested by the ITI consensus report on PROMs by Feine *et al* and Tonetti *et al*^7 8^. Assessments are conducted prior to implant placement, at implant placement, 2 weeks after implant placement, before prosthesis placement, 3 months post-prosthesis placement and at 12 months after prosthetic/denture placement. Both ClinROs and PROMS are assessed at each time point. Figure 1 provides an overview of the data collection schedule.

### Investigator training and calibration

Clinical data collection is conducted by participating clinicians after receiving detailed instruction on project protocols, data collection procedures and registration requirements. This is conducted by the principal investigator (PI) in an one-on-one setting and covers the study workflow, data storage protocols and data security measures. Participating clinicians are taught how to include patients in accordance with the principles of informed consent and how the questionnaires are automatically sent. Additionally, the data collection platform is introduced in detail, and secure user accounts are created to ensure safe access to the system. The platform GEneric Medical Survey Tracker (GemsTracker) has been designed to streamline the process for clinicians, enabling seamless integration during clinical consultation hours.^15^ GemsTracker incorporates two-step verification and is configured to support Dutch, English and Spanish. On completion of this training, practices are ready to begin patient inclusion immediately.

To ensure sustained participation throughout the longitudinal follow-up period, multiple engagement strategies were implemented. These included automated reminder emails and in-appointment prompting by clinical staff.

### Data management

The collected data are securely stored on the servers of Erasmus MC Hospital, in full compliance with local data protection policies and regulations. Data collection is conducted using the GemsTracker platform in both the Netherlands and Spain.

All data are encrypted and anonymised to ensure participant confidentiality. Storage will take place on password-protected servers and computers. Access to both the raw data and the final study dataset is strictly limited to the designated PIs only.

Raw data are used to monitor response and progress of the project. Figures and tables visualising a summary of the collected data are used to regularly inform participating or clinicians about (their contribution to) the project.

Data requests accompanied with a research proposal need to be sent to the PIs of this project.

### Statistical methods

Descriptive statistics are used to describe the composition of the study population and to present collected data to participating dental practices. Patient-reported outcome scores are summarised at the domain level as the mean of corresponding Likert-scale items (range 1–5) per time point, enabling longitudinal monitoring of outcomes across all treatment and follow-up time points. Participating practices and patients receive periodic aggregated data summaries to support engagement and benchmarking.

Psychometric testing of the DENS instrument will take place at the end of the data collection phase in accordance with COSMIN methodology, including assessment of internal consistency (Cronbach’s α), structural validity and construct validity by examining associations between PROM scores and clinical outcome measures and case-mix variables.^16^ Participants with missing data at individual time points are retained in the dataset using available-case analysis. Time points with a response rate below 50% will be flagged and treated with caution in the analyses. All analyses will be conducted using IBM SPSS Statistics V.28.0.1.0.

### Research ethics and governance

This study received ethical approval from multiple institutional review boards: the Medical Ethical Review Committee of Erasmus University Medical Center (MEC-2023-0688, 2 November 2023), Catharina Hospital Eindhoven (2024.076), Anna Hospital Geldrop (MEC-24.008), St. Antonius Hospital (R&D/Z24.070/DENS), University Medical Center Groningen (METc 2024/389) and the Medical Ethical Review Committee of the University of Santiago de Compostela (CEI-SL 2024/122, 3 June 2024). This study is considered low risk with regards to research quality and integrity, as well as ethical considerations related to the rights, safety and well-being of participants, since no additional health-related risks beyond standard care are imposed on them. The clinical data collected in this study are part of the routine treatment protocol. Written informed consent was obtained from the study participants prior to data collection. Participants received a patient information sheet from the clinician describing the study procedures, the purpose of the study, the voluntary nature of participation and data handling practices. All data are pseudo-anonymised before analysis. The investigators declare no financial or competing conflicts of interest.

The study design aligns with ICH Good Clinical Practice (GCP) E6(R3) principles through informed consent procedures, ethical oversight from independent committees and encrypted data management protocols. The implementation of standardised outcome measures, validated instruments with multilingual translation and structured investigator training ensures data integrity and scientific rigour. This proportional, risk-based approach demonstrates flexible GCP application appropriate for non-interventional studies integrated within routine care workflows.

This study protocol was developed in accordance with the Core Outcome Set-STAndardised Protocol Items (COS-STAP) statement, which provides guidance for COS development study protocols.^17^ A completed COS-STAP checklist is included as a supplementary file alongside this manuscript.

### Dissemination policy

This project will promote acceptance for the broad implementation of the DENS through scientific publications, presentations at conferences and engagement with scientific societies. The data will be made available for the validation of the outcome set, the evaluation of current implantology outcomes and for research purposes related to the field of implantology.

Additionally, the tool will be made available for clinical practice, promoting standardised and evidence-based research in implant dentistry.

## Discussion

This protocol describes the development of DENS, a standardised outcome measurement instrument combining ClinROs and PROMs for routine dental implant practice. Within implant dentistry, there is currently no consensus on how to systematically measure and report treatment outcomes, particularly from the patient perspective. This lack of standardisation limits cross-study comparisons, evidence synthesis and the ability to benchmark clinical performance across settings and countries. The DENS addresses this gap by operationalising the mandatory outcome domains of the ID-COSM framework^8^ into a practical, multilingual instrument applicable to both clinical practice and research.

The clinical relevance of systematic PROM collection in implantology is well supported. Patient-reported outcomes have been shown to effectively predict treatment success and identify key risk factors for postoperative complications, including patient-related factors such as age and alcohol consumption, and surgery-related factors such as pain perception and bone augmentation procedures.^18^ Structured collection of these outcomes enables clinicians to identify high-risk patients, support targeted interventions and contribute to evidence-based quality improvement. By enabling international benchmarking of both clinical and patient-reported outcomes, the DENS has the potential to advance implant dentistry through robust comparative research and standardised quality assurance.

The DENS was developed through international collaboration across three academic centres, building directly on the ID-COSM domains rather than initiating a new consensus process, given the rigorous foundation already established by Tonetti *et al*.^8^ Items were drawn from validated instruments, refined through iterative expert working sessions and reviewed by the ID-COSM authors prior to finalisation. Forward-backward translation into Dutch, English and Spanish ensures cross-national applicability and enables outcome comparability across diverse patient populations and healthcare settings.

### Implementation feasibility and longitudinal data collection

The DENS is administered digitally through the GemsTracker platform, which integrates into routine clinical workflows and supports multilingual questionnaire delivery across both countries. A key challenge in longitudinal PROM research is maintaining engagement across multiple time points, as response rates typically decline after initial completion.^19^ This is compounded by differences in healthcare delivery between the Netherlands and Spain: Dutch patients frequently return to their general dentist after implant placement, potentially limiting contact with the implantologist, while in Spain implantologists more commonly perform prosthetic restorations, facilitating continued follow-up. To support sustained participation, the study includes only practices able to follow-up patients for at least 1–2 years and implements automated reminders and in-appointment prompting. The concise questionnaire design and appointment-linked administration are further expected to support adherence. Whether these strategies translate into adequate long-term response rates will be evaluated and reported as part of the planned psychometric validation.

### Cross-cultural considerations

By incorporating both Dutch and Spanish populations from the outset, the DENS was designed with cross-national applicability in mind. Research on international PROM initiatives consistently emphasises that local co-development and culturally adapted communication are essential for successful implementation.^20 21^ The forward-backward translation methodology applied to all DENS questionnaires is intended to ensure linguistic and cultural equivalence. The extent to which this equivalence holds across both study populations will be formally assessed during the psychometric validation phase.

### Study limitations

Several limitations of this study should be acknowledged. Participating practices are selected based on their capacity for longitudinal follow-up and research engagement, which may limit generalisability to less research-engaged clinical settings. The 12-month follow-up captures short-term to medium-term outcomes but does not allow assessment of longer-term implant survival or late complications. The study is currently limited to two countries; further variation in global healthcare systems, particularly in resource-limited settings, may constrain the broader applicability of the implementation strategy. Finally, formal patient and public involvement at the instrument level was not undertaken, though the ID-COSM domains on which DENS is based were developed with explicit patient participation, including focus groups and Delphi involvement of people with experience.^8^ Future iterations of the DENS should incorporate formal Patient and Public Involvement (PPI) at the item level to further strengthen content validity from the patient perspective.

This protocol describes the coordinated, multinational effort to develop and implement the DENS as a standardised outcome measurement instrument for implant dentistry. By systematically capturing both clinician and patient perspectives within routine clinical practice, the DENS provides a foundation for evidence-based benchmarking, quality improvement and future research in implant dentistry.

## Supplementary material

10.1136/bmjopen-2025-108369 online supplemental file 1
