# Supplementary material for: International collaboration to develop a dental implant standard set: study protocol for a multicentre prospective cohort study for the validation of a core outcome set
Source: BMJ Open. 2026 Sep 11;16(9):e108369. doi: 10.1136/bmjopen-2025-108369 (PMC13583730; doi:10.1136/bmjopen-2025-108369)
Supplement: Supplementary file 1 [file bmjopen-2025-108369.reviewer_comments.pdf]

PEER REVIEW HISTORY

BMJ Open publishes all reviews undertaken for accepted manuscripts. Reviewers are asked to complete a checklist review form and are provided with free text boxes to elaborate on their assessment. These free text comments are reproduced below.

ARTICLE DETAILS

Title (Provisional)

An international collaboration to develop a dental implant standard set – study protocol for a multicenter prospective cohort study for the validation of a core outcome set

Authors

Chin, Milton; Pijpe, Justin; Blanco, Juan; Cune, Marco; Wolvius, Eppo B; Kragt, Lea

---

VERSION 1 - REVIEW

**Reviewer**	**1**
**Name**	**Levey, Colin**
**Affiliation**	**University of Dundee, School of Dentistry**
**Date**	**01-Jun-2026**
**COI**	**None**

I welcome the authors desire to improve the methodology of studies investigating the outcomes of implant treatment. Especially in the area of patient experience - which is so important for contextualising care and obtaining consent from patients. Specific questionnaires to identify the particular experience of implant patients are to be welcomed.

However, there are a few areas of this submission which require either further explanation, development or revision.

Given that this is defined as a protocol for the "development" and "vailidation" of the core outcome set, the submission presents an already developed questionnaire and results of early validation work. Either I have misunderstood what this paper is trying to describe, or it requires tightening of the wording to make it clear what this is a protocol for, if indeed it is a protocol at all.

While it is clear that you have taken ID-COSM into careful consideration, including undertaking work with the authors of that Core Outcome Set, it has taken me a while to understand how the presented protocol relates to ID-COSM. Again, I may be mis-reading the submission, but it appears that you are not proposing a new Core Outcome Set (at the level of outcome domains), but instead are proposing a new tool to measure aspects of the ID-

COSM core outcome set. If this is the case, then further information about how you developed the tool (questionnaire) is required. This appears to be a series of discussions with experts in the field, rather than a clear method, which could be reasonably expected to include patients as partners in research. Further, reference to how this relates to the COSMIN framework (https://www.cosmin.nl/) is then needed. Returning to my point about whether this paper is about development of a questionnaire or validation of one, there needs to be clarification - at the moment it seems to be a mixture of the two.

Patient and public involvement - what is described on page 11, line 45 onwards, does not meet the accepted definition of PPI. What is described here is research participation, rather than involvement in design or dissemination. Related to the point about about development of the questionnaire, there would be clear merit in involving patients in drafting the patient questionnaire - to ensure that important areas are covered and that it is readable and easy to complete for a lay audience.

Preliminary results - could this be published as a separate pilot study? It was not clear where you got your initial expected completion rate of 70%, which I thought was quite high. On reading this section, it would appear that overall study completion will be much lower than this (43-45% for staff, 32-35% for patients).

Other figures need to be checked here too - on page 6, line 24 you say that there will be 20 practices from the Netherlands and Spain (total of 40?). Then, on page 12, line 25, you say there will be 15 practices per country. Which is correct? and do you have confidence that this is realistic? any why? The range of patients of 900-1500 is very wide, and taking the lowest number and your response rate at the end, your completion rate will be much closer to 300, rather than the 630 lower estimate on page 12.

Overall, I think that the proposal of developing and validating an implant PROM questionnaire is a good one. However, I do not think that, in its current form, this submission follows current best practice in developing or reporting this type of work.

VERSION 1 - AUTHOR RESPONSE

<u>Reviewer #1</u>

Dr. Colin Levey, University of Dundee

Response: We thank Dr. Levey for their thorough and constructive review. Please see our detailed responses below.

General comment:

> *I welcome the authors' desire to improve the methodology of studies investigating the outcomes of implant treatment. Especially in the area of patient experience — which is so important for contextualising care and obtaining consent from patients. However, there are a few areas of this submission which require either further explanation, development or revision.*

Response: We appreciate the reviewer's positive assessment of the study's aims and their detailed feedback, which we have addressed point by point below.

Comment 1:

> *Given that this is defined as a protocol for the "development" and "validation" of the core outcome set, the submission presents an already developed questionnaire and results of early validation work. Either I have misunderstood what this paper is trying to describe, or it requires tightening of the wording to make it clear what this is a protocol for, if indeed it is a protocol at all.*

Response: We thank the reviewer for this important observation, which highlighted a lack of clarity in our original manuscript. We acknowledge that the terminology was inconsistent and potentially caused confusion. To clarify the correct framing: the DENS questionnaire was developed by our international team, based one core-outcome domains established by the ID-COSM consensus initiative (Tonetti et al., J Clin Periodontol 2023). The current manuscript is a development protocol, describing the rationale, methodological approach, and implementation of the DENS instrument in a prospective multicenter cohort, with aim to conduct formal psychometric validation (internal consistency, structural validity, construct validityof the DENS instrument. We have revised the aim with a clear description of the development phase at line 145-153.

"The aim of this protocol is to describe the development and implementation of the DEntal implaNt Standard set (DENS), a standardized outcome measurement instrument comprising clinician-reported outcomes (ClinROs) and patient-reported outcome measures (PROMs), based on core-domains of the ID-COSM framework, for evaluating peri-implant health and restorative outcomes in routine implant dentistry practice across two countries.

Formal psychometric validation of the DENS instrument is planned as a subsequent step following completion of data collection.

Following psychometric validation, the DENS instrument will be made available to support in clinical practice and research settings internationally."

Comment 2:

> *It appears that you are not proposing a new Core Outcome Set (at the level of outcome domains), but instead are proposing a new tool to measure aspects of the ID-COSM core outcome set. Further information about how you developed the tool (questionnaire) is required. This appears to be a series of discussions with experts in the field, rather than a clear method. Further, reference to how this relates to the COSMIN framework is then needed.*

Response: The reviewer is correct: the DENS does not propose new outcome domains but rather translates the existing ID-COSM mandatory domains into a practical measurement instrument. We have clarified this distinction throughout the manuscript. Regarding the development methodology: we have substantially expanded the Development section (see also Editor Comment 6) to describe the structured process more transparently, including the basis in ID-COSM, the iterative working sessions with the international team, item selection criteria, expert consultation, and translation methodology. We have also added reference to the COSMIN framework to describe how the planned psychometric analyses will assess the validity and reliability of the DENS instrument at line 206-209.

"Formal psychometric validation, including assessment of internal consistency (Cronbach's α), structural validity, and construct validity in accordance with COSMIN methodology, is planned at the end of the data collection phase and will be reported in a separate publication."

Comment 3:

Patient and public involvement — what is described on page 11, line 45 onwards, does not meet the accepted definition of PPI. What is described here is research participation, rather than involvement in design or dissemination.

Response: We thank the reviewer for this important distinction, which we fully agree with. The activities described in the original manuscript. completion of questionnaires and access to the study website, include research participation rather than patient and public involvement (PPI) in the methodological sense.

We have revised the Patient and Public Involvement section accordingly. We acknowledge that formal PPI at the instrument level, such as involving patients in the development and review process, was not part of the DENS development process. We have clarified this in the manuscript and noted it as a limitation, with a recommendation that future iterations of the instrument should include more structured patient involvement.

At the same time, we would like to highlight that the ID-COSM outcome domains on which the DENS is grounded were developed with explicit and rigorous patient involvement. Tonetti et al. (2023) involved 31 people with experience of dental implants in international focus groups across four countries, patient representatives participated in the three-round Delphi survey, and 17 patients contributed to the final consensus meeting and formal vote. The DENS therefore builds on a framework that incorporates the patient perspective at the domain level, even though this involvement took place within the ID-COSM initiative rather than within our study.

We have revised the PPI section to reflect both this context and the limitation at line 302-314, as follows:

"The development of DENS is grounded in a framework that was established with explicit patient involvement. The ID-COSM outcome domains, which form the basis of the DENS instrument, were identified through a rigorous international consensus process incorporating the perspectives of 31 people with experience of dental implants, who participated in focus groups across four countries. Patient representatives further contributed to both the three-round Delphi survey and the final consensus meeting (8).

Within the current study, patients participate as research contributors by completing digital questionnaires at six time points throughout their treatment trajectory, capturing their experiences with implant-supported tooth replacement from pre-treatment through to 12-month follow-up. Participants are introduced to the study by their treating clinicians, who obtain written informed consent prior to inclusion. Study information and findings will be disseminated to participants through the study website (www.densresearch.com) upon completion."

Comment 4:

Preliminary results — could this be published as a separate pilot study? It was not clear where you got your initial expected completion rate of 70%. On reading this section, overall study completion will be much lower (43-45% for staff, 32-35% for patients).

Response: We thank the reviewer for this valuable suggestion and for raising the question about the 70% completion rate assumption. The 70% response rate was based on Meyer et al. (2022), who reported a global mean survey response rate of approximately 70% among both patients and healthcare professionals in surgical research (Meyer 2022). We acknowledge, however, that this figure reflects initial response rates rather than longitudinal completion rates across multiple time points, and that the reviewer is correct that sustained completion rates over the full follow-up trajectory are typically considerably lower. It is also important to note that the study is still ongoing, and many patients have not yet reached their final follow-up appointments.

As the study progresses and more patients complete the full trajectory, these rates are expected to increase.

We fully agree with the reviewer's suggestion that the preliminary results would be more appropriately reported as a standalone pilot publication. This also aligns with the editorial guidance received (Editor Comment 4), and we have therefore removed the Preliminary Results section from the revised manuscript. We intend to report the implementation findings, including longitudinal response rates and feasibility outcomes, in a dedicated pilot paper.

Comment 5:

> *Other figures need to be checked: on page 6, line 24 you say there will be 20 practices from the Netherlands and Spain. Then on page 12, line 25, you say there will be 15 practices per country. Which is correct? The range of patients of 900-1,500 is very wide, and taking the lowest number and your response rate, your completion rate will be much closer to 300 than the 630 lower estimate.*

Response: We thank the reviewer for pointing this out. The target is 15 practices per country (30 in total), as stated in the Sample Size section. The reference to 20 practices was an error and has been corrected. As recruitment was still ongoing at the time of submission, it is possible that the final number may fall short of 30, this has been acknowledged in the revised manuscript.

Regarding patient numbers: with 30 to 50 patients per practice, the expected total ranges from 900 to 1,500, which is indeed a wide range and reflects variation in practice size and patient volume. The reviewer is right that actual completion rates are likely lower than the 70% assumption. This figure was based on Meyer et al. (2022), which reports initial survey response rates rather than longitudinal completion rates, a distinction we now acknowledge in the revised Sample Size section. Given the exploratory nature of this study, no specific completion estimate is presented. Instead, we describe the planned sample as adequate for descriptive analyses and psychometric testing.

Comment 6:

> *Overall, I think that the proposal of developing and validating an implant PROM questionnaire is a good one. However, I do not think that, in its current form, this submission follows current best practice in developing or reporting this type of work.*

Response: We thank the reviewer for this overall assessment. We believe the revisions made in response to the specific comments, in particular the clarification of the development framework, the expanded methods, the reference to COSMIN, the revised PPI section, and the inclusion of the COS-STAP checklist, substantially strengthen the manuscript and bring it in line with current best practice for reporting outcome measurement instrument development protocols.
