# Supplementary material for: International collaboration to develop a dental implant standard set: study protocol for a multicentre prospective cohort study for the validation of a core outcome set
Source: BMJ Open. 2026 Sep 11;16(9):e108369. doi: 10.1136/bmjopen-2025-108369 (PMC13583730; doi:10.1136/bmjopen-2025-108369)
Supplement: Supplementary file 2 [file bmjopen-16-9-s001.docx]

**COS-STAP Checklist**

| **Item** | **COS-STAP item** | **Where in manuscript (page — approximate line)** | **Status** |
| --- | --- | --- | --- |
| **TITLE / ABSTRACT** | | | |
| **1a** | Identify in the title that the paper is a COS development protocol | **Title page – p. 1, line 1-3** | **Applicable** |
| **1b** | Provide a structured abstract | **Abstract — p. 2-3** | **Applicable** |
| **INTRODUCTION** | | | |
| **2a** | Describe background, rationale for developing the COS, reasons needed, potential barriers to implementation | **Background and Rationale — p. 5-6, line 69-109**  Rationale described: lack of standardized outcome measurement, international variability in implant practice, need for PROMs and ClinROs. Barriers addressed in Study Limitations (selection bias, practice variation, longitudinal adherence, global generalizability). | **Applicable** |
| **2b** | Describe specific objectives with reference to developing a COS | **Aim — p. 7, line 113-121**  Aim describes development and implementation of DENS based on ID-COSM domains. Explicitly states the psychometric validation is a subsequent step. Context of use beyond NL/ES mentioned. | **Applicable** |
| **SCOPE** | | | |
| **3a** | Describe health condition(s) and population(s) covered | **Eligibility criteria — p. 12-13, line 217-230**  Population: patients aged 18–75 years with indication for implant-supported fixed or removable prosthesis. Exclusion criteria listed (inadequate oral hygiene, low digital literacy, pregnancy, radiotherapy, language barriers, major bone augmentation). | **Applicable** |
| **3b** | Describe intervention(s) covered | **Clinical-Reported Outcomes; Eligibility criteria — p. 13, line 224-230**  Interventions: single-unit and multi-unit fixed dental prostheses (screw-retained or cemented), removable partial and full dentures. Minor bone augmentation and soft-tissue grafts included. | **Applicable** |
| **3c** | Describe context of use for the COS | **Aim — p. 7, line 113-121; Dissemination Policy — p. 18, line 330-336**  Aim now states DENS is for use in 'routine implant dentistry practice' (clinical and research) and will be made available 'beyond the two participating countries' after validation. Dissemination Policy confirms availability for clinical practice and research. | **Applicable** |
| **METHODS** | | | |
| **4** | Describe stakeholder groups, rationale for involvement, and how individuals are identified | **Establishment of research team — p. 8, line 133-139; Patient and Public Involvement — p. 13-14, line 231-243**  Research team: implantologists, prosthodontists, researchers from 3 academic centres (Erasmus MC, USC, UMCG). ID-COSM authors consulted as external expert stakeholders.  PPI section now explicitly acknowledges that formal patient involvement in DENS design was not undertaken, while noting that ID-COSM domains (on which DENS is based) were developed with 31 People with lived experience in focus groups and patient representatives in Delphi and consensus meeting (Tonetti et al. 2023). | **Applicable** |
| **5a** | Describe information sources used to identify the list of outcomes | **Development of the preliminary DENS — p. 8–9, line 159-187**  Outcome domains: ID-COSM mandatory domains (Tonetti et al. 2023) established via systematic reviews, patient focus groups, 3-round Delphi.  PROM items: adapted from OHIP-14 (10), OHIP-EDENT (9), PIDAQ (12), DSQ (11), PSQ (13). | **Applicable** |
| **5b** | Describe how outcomes may be dropped/combined, with reasons | **Development of the preliminary DENS — p. 8–9, line 151-161**  Item exclusion criteria now described: (1) clinical relevance to routine practice, (2) alignment with ID-COSM domain, (3) comprehensibility and respondent burden. Items considered overlapping, redundant, or insufficiently specific were excluded through team consensus. | **Applicable** |
| **6** | Describe plans for how the consensus process will be undertaken | **Development of the preliminary DENS — p. 8–9, line 140-167**  ID-COSM domains were already established through rigorous international consensus, therefore new domain-level Delphi are not needed. Item selection conducted through structured expert working sessions + ID-COSM author consultation. | **N/A** |
| **7a** | Describe the consensus definition (e.g. pre-specified Delphi threshold) | No formal Delphi was conducted.  Item selection used expert team consensus within pre-defined criteria (see 5b), not a scored Delphi with a threshold. This is methodologically appropriate and the rationale is explained (p. 8-9) | **N/A** |
| **7b** | Describe procedure for adding/combining/dropping outcomes during consensus | **Development of the preliminary DENS — p. 8–9, line 150-160**  Exclusion criteria for items now described (see 5b). Items dropped through team consensus based on overlap, redundancy, or insufficient specificity. | **Applicable** |
| **ANALYSIS** | | | |
| **8** | Describe how outcomes will be scored/summarised; how participants receive feedback | **Statistical Methods — p. 16, line 291-304**  Scoring: PROM scores at domain level as mean of Likert-scale items (range 1–5) per time point.  Feedback: participating practices and patients receive periodic aggregated data summaries for benchmarking.  Psychometric testing: COSMIN methodology described (Cronbach's α, structural validity, construct validity). | **Applicable** |
| **9** | Describe how missing data will be handled | **Statistical Methods — p. 16-17, line 301-304**  Missing data approach with an available-case analysis. Time points with response rate below 50% flagged and treated with caution. Engagement strategies to limit dropout mentioned (reminders, in-appointment prompting). | **Applicable** |
| **ETHICS AND DISSEMINATION** | | | |
| **10** | Describe ethics approval plans and how informed consent is obtained | **Research Ethics and Governance — p. 17–18, line 307-331**  IRB approvals listed with reference numbers and dates (MEC-2023-0688; 2024.076; MEC-24.008; R&D/Z24.070/DENS; METc 2024/389; CEI-SL 2024/122).  Written informed consent by treating clinician described. Patient information sheet mentioned. | **Applicable** |
| **11** | Describe plans to communicate results to participants and COS users | **Patient and Public Involvement — p. 13-14, line 231-243; Dissemination Policy — p. 18, line 330-336**  Study findings disseminated via study website (www.densresearch.com) to participants. Scientific dissemination via publications, conferences, scientific societies. DENS tool made publicly available for clinical practice and research. | **Applicable** |
| **ADMINISTRATIVE INFORMATION** | | | |
| **12** | Describe funding sources and role of funders | **Funding statement — p. 23, line 486-487**  **Acknowledgements — p. 23, line 404-409**  ITI grant 1661-2022. Funders not described as having a role in design or analysis | **Applicable** |
| **13** | Describe potential conflicts of interest and how managed | **Competing Interests — p. 23, line 488**  No conflicts of interest declared. | **Applicable** |

*From: Kirkham JJ, Gorst S, Altman DG, et al. (2019) Core Outcome Set-STAndardised Protocol Items: the COS-STAP Statement. Trials 20, 116. https://doi.org/10.1186/s13063-019-3230-x*
